# Barriers, Challenges, and Facilitators Experienced by Healthcare Professionals in Providing Immediate and Efficient Care in the Emergency Department: A Mixed-Methods Study

**DOI:** 10.7759/cureus.86089

**Published:** 2025-06-15

**Authors:** Anchal Gupta, Sandeep Arya, Pooja Yadav, Anjali Gupta, Ankit Raj, Tarangini Maurya, Pawanpreet Kaur

**Affiliations:** 1 Medical Surgical Nursing, College of Nursing, King George's Medical University, Lucknow, IND; 2 Mental Health Nursing, Faculty of Nursing, Uttar Pradesh University of Medical Sciences, Saifai, IND; 3 Critical Care Nursing, Charing Cross Hospital, Imperial College Healthcare NHS Trust, London, USA; 4 Maternal and Child Health Nursing, College of Nursing, King George's Medical University, Lucknow, IND; 5 Community Health Nursing, Faculty of Nursing, Uttar Pradesh University of Medical Sciences, Saifai, IND; 6 Medical Surgical Nursing, Faculty of Nursing, Uttar Pradesh University of Medical Sciences, Saifai, IND

**Keywords:** challenges faced, communication barriers, emergency department (ed), healthcare delivery, healthcare professionals (hcp), nursing, safety concerns, staffing shortages, triage protocols, barriers

## Abstract

Background: Emergency departments (EDs) are frontline units serving 24×7 services to patients. Healthcare professionals often face several barriers and challenges that hinder them from delivering immediate and efficient care. Overcrowding, inadequate staffing, lack of triage skills, communication gaps, and workplace aggression from relatives of patients are the major factors that impact ED performance. Identifying these barriers, challenges, and facilitators is important for quality emergency care services.

Materials and methods: A sequential explanatory mixed-method design was employed in the ED of Uttar Pradesh University of Medical Sciences, Saifai, India. The study was divided into two phases. Phase I used a quantitative descriptive approach with a reliable Likert-scale questionnaire to estimate barriers and facilitators among 53 participants (22 doctors and 31 nurses). Phase II used a qualitative approach through semi-structured interviews with 13 purposively selected participants until data saturation was achieved. Qualitative data were analysed through thematic analysis.

Results: Quantitative findings showed that 26 (49.1%) participants marked inadequate staffing, 32 (60.4%) identified delayed information transfer, and 42 (79.2%) showed inefficient triage as primary barriers. Facilitators reported included team support (n=31, 58.5%), clear protocols (n=32, 60.4%), and continuous professional development (n=31, 58.5%). Qualitative analysis revealed six themes: overcrowding, challenges with triage, safety concerns due to aggressive relatives, shortage of staff, communication barriers, and time-consuming duplication of paperwork. These challenges lead to staff burnout, delayed patient care, and medical errors.

Conclusion: The study critically analysed the barriers, challenges, and facilitators affecting the ED’s performance. It highlights that supportive teamwork, training, and clear communication are key factors in facilitators. Urgent interventions such as improving staff numbers, enhancing triage training, and streamlining administrative processes are important. These issues should be assessed to improve patient outcomes, healthcare staff wellbeing, and the efficiency of emergency services.

## Introduction

The emergency department (ED) is considered the first point of contact for patients seeking urgent medical care. It runs 24×7 and is equipped in such a way to address medical emergencies, including trauma, cardiac problems, respiratory distress, and serious infections [1]. ED staff play an important role in providing quality care to patients. However, several barriers, challenges, and facilitators influence the capacity to work efficiently.

A major barrier to performance is overcrowding in the ED [2]. This occurs due to a mismatch between the number of people who require emergency care and the capacity of the hospital. A large number of cases are being treated in the emergency room, including medical and surgical, urgent and even non-urgent, and this is the root cause of congestion [3]. Other factors such as patient overload, inefficient triage practices, non-availability of beds, variations in staff experience, and quality of training also contribute to the problem [4-6]. Delays in internal and external patient transport further exacerbate the problem [7].

From the perspective of nurses, arguments and conflicts with the relatives of patients regarding the care, as well as within the teams, can lead to delays in the efficient care in the ED. These interactions occasionally take the form of workplace violence [8]. A lack of basic comprehension of triage and incompetence in the utilization of triage also jeopardize the immediate care [9]. As the ED is an unpredictable zone where diverse patients with unpredictable disease scenarios are reported, it is important that skilled healthcare professionals should be placed here [10]. Studies report that nurses' lack of knowledge or staged formal training leads to the patient being over-triaged or inaccurately triaged in the ED. Triage nurses reportedly put patients lower or higher than their actual levels, according to a study by Suamchaiyaphum et al. [11].

Facilitators such as supportive team culture, delivering excellent patient-centered care, and professional development opportunities improve emergency care [12,13]. Nurses report that a lack of knowledge and competency is also a significant barrier. In contrast, doctors cite time constraints and lack of health promotion infrastructure in the ED as major challenges to intervention delivery. Paramedics staff report insufficient training as a critical limitation [12,14]. A prior study showed three major themes that reflect the systemic and practical challenges faced by emergency staff like environmental and systemic challenges that increase safety risks, the importance of team work in managing the patients, which is hampered by hierarchical structure and professional silos, and difficulty of delivering high quality care to a population of patients who are marginalized, while addressing safety threats [15]. 

The present study aimed to assess the barriers faced by healthcare professionals in delivering immediate and efficient care, identify the challenges, and recognize the facilitators that enable in provision of prompt care in the ED. 

## Materials and methods

Study design and setting

The study employed an explanatory, sequential mixed-method design, which was conducted in two phases. Phase I included the quantitative component using a descriptive approach, while Phase II included the qualitative components. The study was conducted in the ED of the Uttar Pradesh University of Medical Sciences (UPUMS), Saifai, India. The hospital is a tertiary care facility and receives a wide variety of cases, and is equipped with multifunctional aids for patient care. This study focuses on three key components: barriers, challenges, and facilitators. Barriers and facilitators were analyzed through Phase I (quantitative) while challenges were explored in Phase II (qualitative).

Eligibility

A convenience sampling technique was used for data collection based on predefined inclusion criteria, i.e., healthcare professionals (doctors and nurses) working in the ED with more than one year of experience and willing to participate in the study. Exclusion criteria included paramedics, allied healthcare professionals, and those already involved in similar studies during the time of data collection period. 

Data collection

Data were collected separately from the doctor and nurse participants. The researcher received the consent letter and explained the purpose of the study, following which a questionnaire (Tools 1, 2, 3; see Appendices) was given for analysis of barriers and facilitators. Phase II of the study involved qualitative analysis through purposive sampling to create a subset of doctors and nurses’ participants for the generation of themes. Scheduled dates were finalized for face-to-face interviews with participants. The same participants from the quantitative phase also took part in the qualitative phase.

Initially, 20 participants (10 doctors and 10 nurses) were invited; however, data saturation was achieved after interviewing the 13th participant (six doctors and seven nurses). The participants were allowed to choose a convenient time and location for the interviews. It nearly took 20-25 minutes to finish each interview. In order to maintain the richness of the data, maximum variation techniques were utilized based on their questionnaire response on years of experience in the ED and responses regarding barriers and facilitators.

Study tools

For quantitative analysis, a pre-tested, semi-structured questionnaire (Tool 2, see Appendices) using a five-point Likert scale was formulated to assess the barrier and facilitator components. The tool reliability (Tool 2 and Tool 3, see Appendices) was calculated through Cronbach's α: r=0.73 for barriers to immediate and efficient care tool, r=0.88 for facilitators. Content validity of the tool was established by consulting with experts in the medical and nursing fields. The questionnaire used in the quantitative assessment served as a guide for the interviewer. Questions were rephrased to explore the participants' responses in greater depth. To get further information from the participants, follow-up prompts such as “What do you mean?” and “Can you expand further?”

Study process

All the participants were first contacted for Phase I of the study. After explaining the purpose of the study, consent was taken from participants, and each participant was given a unique serial number. The researcher used the unique serial number for identification in both phases.

In Phase II, two groups were formed: Group 1 (doctors) and Group 2 (nurses). Four researchers were allocated to conduct the interviews, with two researchers in each group; one researcher took notes, and the other researcher asked the questions. In order to maintain uniformity of the group, an effort was made to maintain gender balance among researchers. A discussion was scheduled among the researchers prior to data collection to enhance understanding regarding qualitative research and to ensure that all researchers were in accord. The time and place were decided by the participants. Each interview lasted approximately 20-25 minutes. Additionally, field notes taken during data collection were used further for data analysis. Throughout the process of the interview, participants were given positive encouragement to speak openly.

Statistical analysis

After data collection, a master sheet was prepared in MS Excel software version 2019 (Microsoft Corporation, Redmond, Washington, United States) to record the responses of the participants. IBM SPSS Statistics for Windows, version 20 (Released 2011; IBM Corp., Armonk, New York, United States) was used to analyze the quantitative data. The quantitative data were manually noted by the authors during the interviews on rough sheets. These notes were read multiple times to ensure familiarity with the content. After this process, similar responses were grouped and analyzed to identify recurring themes, which were then coded (e.g., Theme 1, Theme 2, Theme 3, and so on) accordingly. Initially, there were 13 codes; some codes were similar and, hence, they were finally grouped into six themes. The content was analyzed manually, and themes were generated and interpreted.

Ethical consideration

The study was approved by the Research Cell, Uttar Pradesh University of Medical Sciences (approval number: 230/RC/UPUMS/2023-24). After obtaining ethical clearance, data were collected from the participants with their endorsement of consent.

## Results

Quantitative components

Table 1 gives the demographic profile of the study participants. A total of 53 individuals participated, including 31 nurses and 22 doctors. Among them, 29 (54.7%) were male and 24 (45.3%) were female. The majority of the participants (n=26, 49.1%) had 5-10 years of experience.

**Table 1:** Distribution of participants based on demographic characteristics (N=53)

Demographic Profile	Frequency (Percentage)
Sex	
Male	29 (54.7%)
Female	24 (45.3%)
Age group (years)	
20-30	12 (22.6%)
30-40	26 (49.1%)
40-50	11 (20.8%)
>50	4 (7.5%)
Experience (years)	
1-3	13 (24.5%)
3-5	12 (22.6%)
5-10	24 (45.3%)
>10	4 (7.5%)
Professional role	
Doctor	22 (41.5%)
Nurse	31 (58.5%)

**Figure 1:**
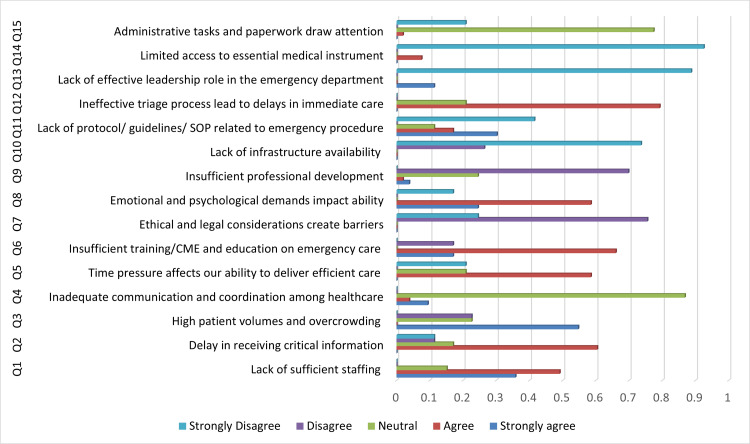
Barriers to immediate and efficient care as reported by the study participants SOP: standard operating procedure; CME: continuing medical education

Figure 1 represents participants’ responses regarding barriers faced by healthcare professionals in providing immediate and efficient care. The majority (n=26, 49.10%) of professionals agreed that a lack of sufficient staff hinders their ability to deliver immediate care. Additionally, 32 (60.40%) participants acknowledged that delays in receiving critical information impacted immediate care. A majority (n=46, 86.80%) remained neutral toward the role of inadequate communication and coordination among healthcare team members in hindering immediate care. Thirty-one (58.50%) participants identified time pressure as a barrier, while 35 (66%) agreed that insufficient training, continuing medical education (CME), and emergency care education created difficulty in delivering immediate care. Regarding ethical and legal considerations, 40 (75.50%) disagreed that this was a barrier to immediate care. Similarly, 37 (69.80%) did not agree that a lack of infrastructure affected care delivery. Moreover, 22 (41.50%) strongly disagreed that the absence of protocols, guidelines, or standard operating procedures (SOPs) for emergency procedures impacted care. Conversely, 42 (79.20%) agreed that an inefficient triage process led to delays in immediate care. The majority of participants disagreed that a lack of effective leadership in the ED and limited access to essential instruments hindered care. Furthermore, 41 (77.40%) had a neutral response toward administrative tasks and paperwork, diverting attention from immediate care.

**Figure 2:**
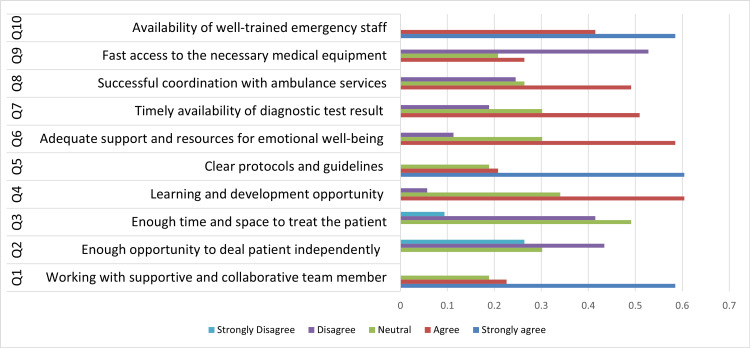
Facilitators for immediate and efficient care as reported by study participants (N=53)

Figure 2 illustrates the key facilitators of immediate and efficient care. The majority of participants (n=31, 58.50%) strongly agreed that working within a supportive and collaborative team enhanced care delivery. However, 23 (43.40%) disagreed with the statement that having enough opportunity to deal with patients independently facilitated efficient care. Additionally, 26 (49.10%) had a neutral response regarding the availability of sufficient time and space to improve patient care quality. A significant number (n=32, 60.40%) agreed that learning and development opportunities contributed to immediate and efficient care, whereas an equal percentage strongly agreed that clear protocols and guidelines enhanced care provision. Furthermore, 31 (58.50%) believed that adequate support and resources for emotional well-being improved care efficiency. Timely availability of diagnostic test results was considered a facilitator by 27 (50.90%) participants, and 26 (49.10%) agreed that successful coordination with ambulance services supported immediate care. Twenty-eight (52.80%) agreed that rapid access to necessary medical equipment enhanced efficient care. Lastly, 31 (58.50%) strongly agreed that the presence of well-trained emergency staff was crucial for enhancing immediate care.

Qualitative components

To assess the challenges faced by healthcare workers working in the emergency department in providing immediate and efficient care, two groups of doctors and nurses were formed, and two researchers were assigned to each group. It was seen that the one-on-one interview with doctors and nurses generated several themes; a total of six themes were finalized through mutual content analysis of focused group discussions.

Theme One: Overcrowding

Doctors and nurses viewed high patient volume as one of the major challenges. High patient volume decreased the quality of care and increased the use of time on non-urgent cases and dissatisfaction among patients. As one doctor noted, “Non-urgent cases are referred to the emergency ward, which has to be segregated, sometimes taking up bed availability.” A nurse stated, “Patients are often accompanied by relatives in the wards, which is not controlled by security guards, creating more overcrowding.” Another added, “Some patient demands special attention even if the situation is not serious.” The majority of participants reported that both urgent and non-urgent cases are referred to the emergency unit. Non-urgent cases not only consume the time of staff but also reduce the bed availability for urgent cases. Relatives of patients often show a lot of aggression, which is not controlled.

Theme Two: Understanding Triage

Participants explained that the triage is quite difficult in a stressful environment. Few reported that a lot of time is consumed in transport activity, and nurses who lack experience are not able to perform triage properly. Senior nurses reported that young healthcare workers, especially nurses, should be properly skilled in triage. A doctor stated, “It is not a stressful environment, but I wonder whether priority is correct, but I am not able to think as there are many patients to deal with.” Another added, “Although doctors do give a provisional plan for most cases, nurses will not perform.” A nurse noted, "We are actually seeing the patients fully, but clearly, the more time consumed at the beginning, the more it takes to perform triage; the entire process of triage is delayed” and “Mostly, the triage is performed by doctors, but a few times the doctor are busy, and then, nurses do triage, which is either over-triage or under-triage by less experienced nurses.”

Theme Three: Safety Concerns

The third theme, Safety Concerns, highlighted that patients are often accompanied by more relatives. Many relatives of patients believe it is the incompetence of doctors that made them unable to resuscitate their patient. A nurse added, "The patient’s relatives are often not manageable by security guards, sometimes displaying aggressive behaviour and not ready to listen." Another nurse shared, “A patient's relative threatened to report me just because I asked them to sit for a while so that I could attend to some other critical patient.” A doctor mentioned, “It is extremely difficult to focus in an environment where a patient's relative is yelling.”

Theme Four: Shortage of Staff

Participants explained that the increased workload due to the shortage of staff impacts the quality of care and often delays critical care cases. A shortage of staff leads to physical and mental exhaustion. Understaffing may result in burnout and higher chances of missed errors. A doctor explains, “I often need to work double shifts to cover the gaps, which impact my efficiency. During peak hour, it is challenging to work with a few staff.” A nurse mentioned, “The present staffing situation focuses on prioritizing tasks; with few members, small emergencies can turn into crisis situations.”

Theme Five: Communication Barriers

Participants who do not speak the local language found it difficult to understand patients. As the ED is a fast-moving environment, it becomes difficult to take action in such a scenario. Participants further added that proper handover was extremely important. It becomes difficult when critical information is not documented, which often creates confusion. A nurse commented, “Patient information was not documented, not even communicated during shift change; I would have made a mistake if I had not asked.” A doctor emphasized, “We need to adopt standard communication techniques and train all staff, as in a high-pressure situation, clear communication with team members can be challenging.”

Theme Six: Administrative Tasks

Participants explained that apart from patient care, they are required to do manual entry of patient records, and some entries are done in an online portal. Time spent on digital and paper documentation often led to reduced interaction with patients. There are compliance requirements toward proper record keeping, which often direct toward documentation. A nurse reported, “We often spend a lot of time updating electronic medical records and documenting patient files, which reduces our availability time for bedside care.” A doctor added, “I have to review documentation. During a peak time like inspection, it is important to send these documents; it takes more hours.”

## Discussion

Although many studies have been conducted, there continues to be a high failure rate in translating evidence into practice. Hence, it is necessary to assess the barriers, challenges, and facilitators. This study found the key barriers endured by healthcare professionals in providing immediate and efficient care, including a shortage of staff, delays in transmitting critical information, time pressure, inadequate training, and ineffective triage systems. The majority of participants disagreed that ethical concerns, infrastructure, or SOPs hinder care. Prior studies suggest that an increased nurse-to-patient ratio improves patient outcomes, whereas reduced staffing numbers lead to a higher number of patients leaving without being seen and further increase the wait times [16-18]. A study by Aloyce et al. in Tanzanian Hospitals found that the majority of triage nurses lacked adequate knowledge of triage, especially in prioritization of patients, which either resulted in a higher or lower level than their actual status. Nurses kept 25% of patients on the lower level and 42% of patients on the higher level of triage [19]. Similar to the study result, it was found that emergency physician has an ethical and legal obligation to assess and stabilize patients with their bodily function [20,21].

In this study, respondents agreed that teamwork, learning opportunities, clear protocols, emotional support, and well-trained staff serve as a facilitator that enhances emergency care delivery. Similarly, other studies suggest a positive association between nursing teamwork and patient-centered care in comparison with poor teamwork [22-25].

Qualitative analysis led to the generation of six key themes affecting emergency care delivery, i.e., overcrowding, triage challenges, safety concerns, staff shortage, communication barriers, and time-consuming administrative tasks. According to participants, overcrowding results from referrals of both urgent and non-urgent cases, leading to strained resources, increasing staff burnout, and medical errors. Other studies also show that referring non-urgent cases leads significantly to overcrowding [24,25]. Triage difficulties arise from stressful environments and inexperienced nurses, leading to delays [11,26]. Bijani and Khaleghi showed that knowing triage protocols, and clinical knowledge are important factors in prioritizing the patients accurately [27]. Moreover, safety concerns arise from aggressive relatives of patients, making it hard for healthcare professionals to focus on patient care. Staff shortages contribute to increased workload, burnout, and delayed critical care [28,29]. Studies support that the administrative challenges, such as a shortage of staff, contribute to a decrease in the efficiency of care. Communication barriers, especially language differences and poor documentation, lead to confusion and potential errors. Time-consuming administrative tasks sometimes divert attention from patient care [30]. Addressing these challenges is important for enhancing emergency care services. 

Limitations

The study explored the barriers and facilitators aspects through quantitative analysis and the challenges through qualitative analysis. This mixed-approach study was conducted in a single centre, focused on the barriers, challenges, and facilitators experienced by healthcare professionals, and gave a valuable insight, but is not without limitations. The main limitation lies in its narrow scope, as it was conducted from a single healthcare facility with few participants. Despite these issues, the study highlights real-world issues such as staff burnout, resource limitations, and communication gaps, as well as facilitators like teamwork, leadership support, and continuous training.

Recommendations

Future research studies should be conducted in multiple centres in diverse capacities (e.g., urban vs. rural, public vs. private) to produce a range of experiences and improve the generalizability of findings. Based on identified challenges, targeted training programs focusing on triage efficiency, management of resources, and inter-professional communication should be developed and updated regularly. A study on debriefing sessions and feedback mechanisms for staff to propose practical solutions and their impact on improvement on challenges faced in EDs can be useful.

## Conclusions

This study provides a valuable insight into the barriers, challenges, and facilitators experienced by healthcare professionals in delivering efficient care in the ED. Quantitative results showed that a staff shortage, delays in receiving critical information, time pressure, and lack of proper training are major obstacles. At the same time, supportive teamwork, clear protocols, and ongoing learning opportunities are key to efficient care. The qualitative components of the study deepen these findings by showing systemic issues such as overcrowding, poor triage skills due to inexperience, safety concerns from aggressive patient relatives, communication breakdowns, staff shortages, and time-consuming administrative tasks. The challenges lead to poor quality of emergency care and professional burnout. Conversely, the presence of well-trained staff and effective team collaboration serves as a strong facilitator. The study also highlights the need for improvement in staffing, training in triage protocols, communication, and administrative workflows. Overall, the findings show the urgent need for systemic and targeted interventions to enhance emergency care efficiency and support healthcare workers.

## Figures and Tables

**Figure 3:**
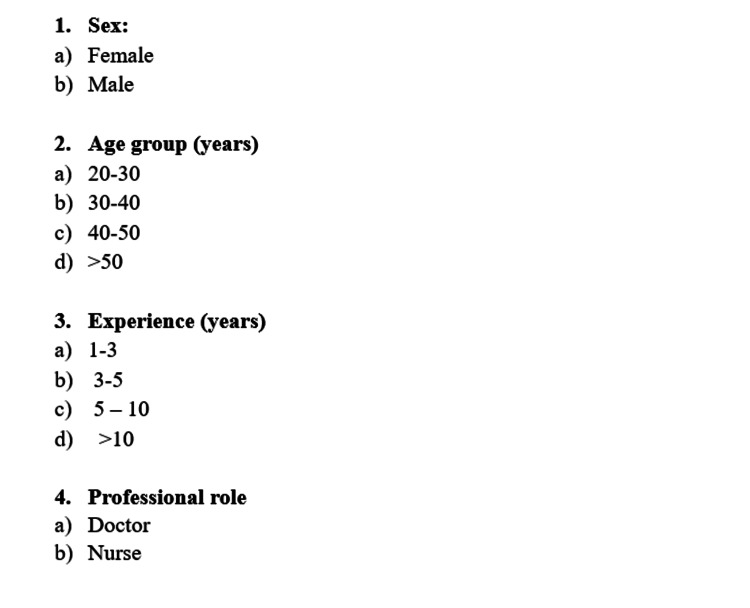
Tool 1: Demographic Profile

## Appendices

Questionnaires

**Table 2:** Tool 2: Barriers to Immediate and Efficient Care *Please rate the following statements on a Likert scale (1 = Strongly Disagree, 5 = Strongly Agree)

Sr. no	Statement*	Strongly Disagree 1	Disagree 2	Neutral 3	Agree 4	Strongly Agree 5
1	Lack of sufficient staffing					
2	Delay in receiving critical information					
3	High patient volumes and overcrowding					
4	Inadequate communication and coordination among healthcare team members					
5	Time pressure affects our ability					
6	Insufficient training/CME and education on emergency care.					
7	Ethical and legal considerations create barriers					
8	Emotional and psychological demands impact our ability					
9.	Insufficient professional development					
10.	Lack of infrastructure availability					
11.	Lack of protocol/ guidelines/ SOP					
12.	Ineffective triage process lead to delays in immediate care.					
13.	Lack of effective leadership role in the emergency department					
14.	Limited access to essential medical instrument					
15.	Administrative tasks and paperwork draw attention					

**Table 3:** Tool 3: Facilitators of Immediate and Efficient Care *Please rate the following statements on a Likert scale (1 = Strongly Disagree, 5 = Strongly Agree)

Sr. no	Statement*	Strongly Disagree 1	Disagree 2	Neutral 3	Agree 4	Strongly Agree 5
1	Working with supportive and collaborative team member					
2	Enough opportunity to deal patient independently					
3	Enough time and space to treat the patient					
4	Learning and development opportunity					
5	Clear protocols and guidelines					
6	Adequate support and resources for emotional well-being					
7	Timely availability of diagnostic test result					
8	Successful coordination with ambulance services					
9	Fast access to the necessary medical equipment					
10	Availability of well-trained emergency staff					
